# The transformative impact of ultra-rapid nanopore sequencing in precision medicine

**DOI:** 10.3389/fimmu.2026.1861975

**Published:** 2026-09-03

**Authors:** Sarah Grasedieck, Brendan J. Keating, Stephen Yip, James Lan, Karen R. Sherwood, Paul A. Keown

**Affiliations:** 1 Department of Medicine, University of British Columbia, Vancouver, BC, Canada; 2 Department of Surgery, University of Pennsylvania, Philadelphia, PA, United States; 3 Molecular Oncology, British Columbia (BC) Cancer, Vancouver, BC, Canada; 4 Department of Pathology and Laboratory Medicine, Faculty of Medicine, University of British Columbia, Vancouver, BC, Canada

**Keywords:** adaptive sampling, genetic testing, genome medicine, infectious disease management, pharmacogenomic (PGx) testing, public health, rare disease diagnostics, transplantation

## Abstract

Nanopore sequencing has emerged as a transformative technology, offering unprecedented speed, ultra-long reads, and portability at a comparatively low cost. These attributes are fundamentally reshaping clinical diagnostics and personalized medicine. This review synthesizes the technological advancements driving nanopore sequencing and elucidates its pivotal applications in time-sensitive areas such as HLA matching in transplantation, infectious disease management, pharmacogenomics, and oncology. Nanopore sequencing also enables comprehensive characterization of immune-related genomic variation including HLA, KIR, LILR, Fc receptor loci, immune regulatory variants and epigenetic features. We explore the profound impact of real-time genomics on patient care, emphasizing the potential for point-of-care testing, the acceleration of clinical decision support, and the realization of precision medicine.

## 1 Introduction

**Figure 1:**
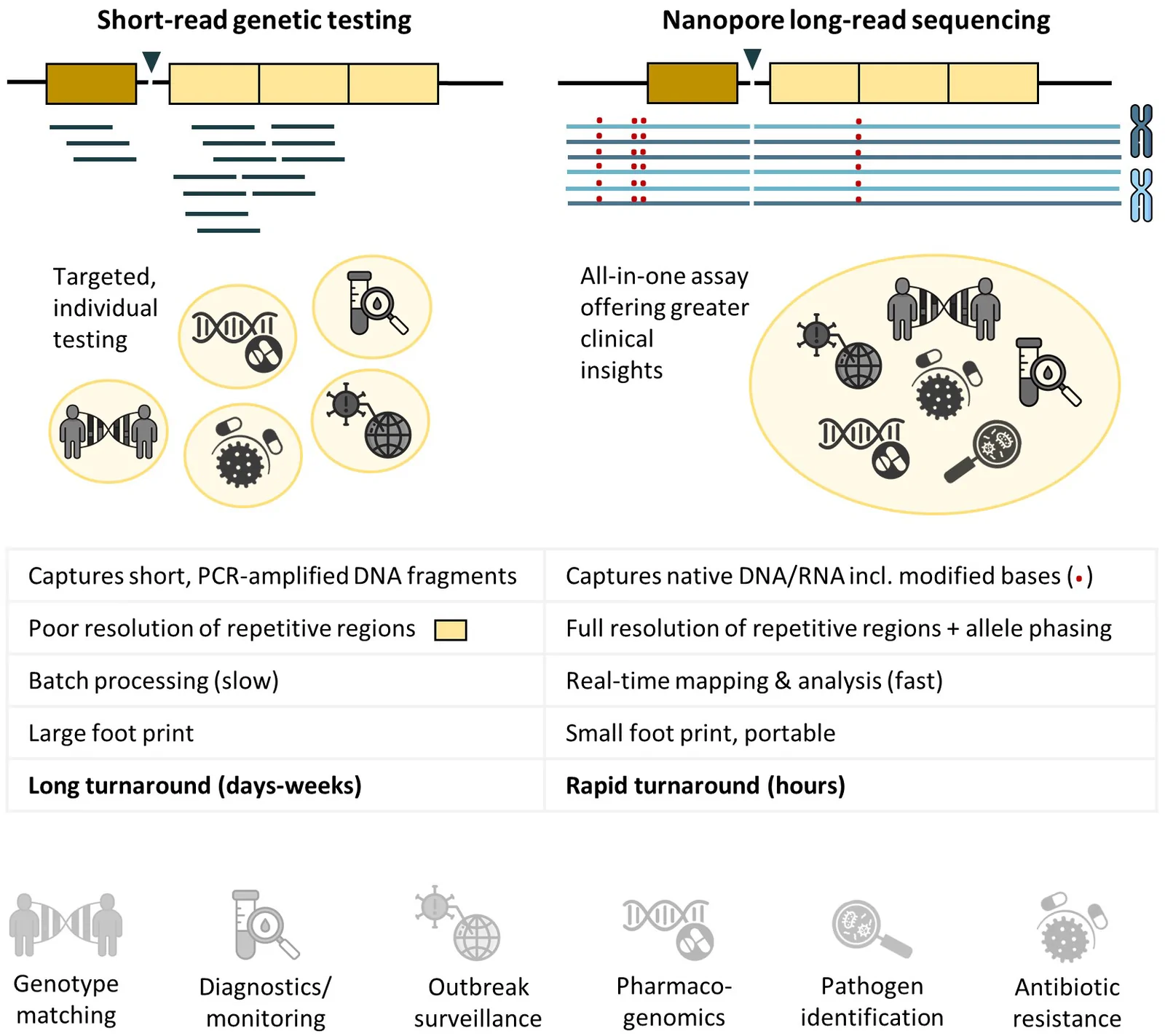
The advantages of nanopore long-read sequencing over traditional, short-read genetic testing.

The landscape of genomic medicine has been historically shaped by Sanger sequencing and, more recently, by second-generation sequencing (formerly next generation sequencing or NGS) platforms (1). These techniques have greatly expanded our knowledge of (epi)genome structure, function, and disease associations (2). While NGS technologies have significantly increased throughput and accuracy while reducing costs over time, their limitations, including short reads (150-300bp) and lengthy turnaround times, have constrained their application in time-sensitive clinical scenarios (**Figure 1**).

Oxford Nanopore Technology (ONT) sequencing is a third-generation sequencing technology where single-stranded DNA or RNA molecules pass through a protein nanopore which induces specific current changes that are directly translated into sequence information (3). This unique detection principle in combination with ONT’s real-time data streaming capacities overcomes critical clinical limitations of other sequencing approaches (**Table 1**), reducing the total assay, and therefore, turnaround time to only few hours. In addition, ONT sequencing enables the real-time capture of native nucleic acid modifications such as 5-(hydroxy)methyl-Cytosine or N^6^-methyl-Adenosine (5(h)mC or m6A), investigation of chromatin conformation and RNA poly(A) tail length, supports a high throughput of up to 1.9Tb per run, and has ultra-long read capabilities, reaching into the megabase (Mb) scale (4–6).

**Table 1:** Main characteristics of current sequencing platforms.

	Illumina	PacBio (HiFi)	Oxford Nanopore	Roche SBX	Ion Torrent	BGI/MGI
Read Length	50–300 bp (paired-end)	1–25 kb	Up to 2+ Mb, avg. 10-200 kb	up to 1500bp, avg. 300–700 bp	Up to 600 bp	50–300 bp (paired-end)
Read Quality	High (>99% Phred Q>30)	High (>99% Phred Q>30)	Moderate (>99% Phred Q>20, improving)	expected to range from Q20 to Q30	Moderate-high (99% Phred Q20-Q30)	High (>99% Phred Q>30)
Requires pre-amplification	yes	yes	no	yes	yes	yes
Minimum Turnaround Time (variant calling)	24-36 hrs	48-72 hrs	~4 hrs	~8-24 hrs (claimed)	~10-24 hrs	~24 hrs
Cost per Sample	$250 (batched) - $2000	$500 (batched) - $3000	$150 (batched) - $1200	estim. ~$1000/sample with batching reducing costs	$150 (batched) - $1000	$150 (batched) - $1000
Main application focus	WGS, RNA-seq, targeted panels	WGS, de novo assembly, SV/CNV analysis, discovery, Iso-seq, metagenomics	WGS, rapid deployment, full-length RNA-seq, SV/CNV, metagenomics	fast turnaround short-read seq, WGS, RNA-seq	Targeted / amplicon panels, low-plex metagenomics	WGS, RNA-seq, targeted panels, metagenomics, high throughput applications
Breadth of Data	Either genomic or transcriptomic (cDNA), some epigenomic (simultaneous 5mC capture possible)	Either genomic or transcriptomic w isoforms (cDNA), simultaneous base modifications	Either native DNA or RNA w isoforms + base modifications (5mC, 5hmC, 6mA, m6A), proteomics in development	Primarily genomic with limited direct transcriptomic or epigenomic applications known as of now	Genomic or transcriptomic (cDNA)	Genomic or transcriptomic (cDNA)
Strengths	Accurate, scalable, cost-effective, wide variety of analysis tools, extensive support	Long/accurate reads w direct isoform and methylation detection	Fast, longest reads, portable, flexible, real-time analysis, no amplification or copy bias, extensive support, highest information density/run	Best accuracy and throughput at shorter read lengths but capture of intermediate-length reads possible	Fast, simple, cost-effective, suitable for clinical diagnostics	Cost-effective, competitive to Illumina
Weaknesses	Short reads limit SV/phasing, reference dependent, slow, expensive if not scaled	High cost, slow, requires high input	High computational resource demand (data storage & analysis), lowest read accuracy	Cost, limited scalability, platform legacy (still a young technology)	Short reads, lower accuracy for GC-rich regions	Same weaknesses as illumina + fewer third-party protocols / compatibility

In its infancy, ONT sequencing could not compete with other platforms in terms of accuracy and amount of reads per flow-cell, however, recent developments in nanopore flow-cell technology and improved base-calling algorithms have improved precision to a degree that is now suitable for single nucleotide variant (SNV) calling and genome assembly, making ONT sequencing a powerful tool for precision -omics approaches. Recently, another nanopore-based sequencing platform, Roche’s sequencing by expansion (SBX), has entered the field - a first published study in a critical care setting promises rapid and precise results (7). This review aims to provide a comprehensive overview of the technological advancements in nanopore sequencing and their impact on clinical medicine, with a particular focus on transplantation, infectious diseases, pharmacogenomics (PGx), oncology, and other critical applications where speed, accuracy, and accessibility are paramount. Representative landmark studies illustrating the clinical implementation and performance of nanopore sequencing across the major application areas discussed in this review are summarized in **Supplementary Table 1** to provide additional context regarding study design, cohort size, and performance metrics.

## 2 Technological advances in nanopore sequencing

### 2.1 Speed and precision improvements

Compared to short-read (typically up to 300bp) and PacBio long-read sequencing (up to 10kb), early ONT sequencing protocols demonstrated higher error rates, particularly in homopolymer-rich regions (carrying repeated identical bases) and in regions with high GC content as these can lead to speed irregularities of the motor protein while ratcheting the nucleic acid strand through the nanopore (4). Over the last years, ONT sequencing has witnessed continued advancements in both precision and speed. This acceleration is crucial in time-sensitive clinical scenarios, such as infectious disease diagnostics and critical care, where rapid identification of pathogens or genetic mutations can significantly impact patient management. Specific advances include an improved flow cell design and assay chemistry (8), incorporation of deep learning, including transformer models and bi-directional recurrent neural networks (RNNs) into base-calling algorithms, direct data streaming into ONT’s pre-configured analysis platform *Epi2me*, and “Duplex” sequencing, which reads both template and complement DNA strands. In combination, these improvements now achieve median read accuracies as high as Q32 (99.93%) (9). Comprehensive reviews describing the evolution of nanopore sequencing chemistry, hardware, and analytical methods have been published elsewhere (4, 10, 11) and are beyond the scope of this review. As of 2025, ONT has started to promote a routine, scalable 24-hour sample-to-variant human whole genome sequencing workflow for clinical research, with integrated analysis pipelines and PromethION-based high-throughput setups (12).

### 2.2 Read length

One of the most significant advantages of ONT sequencing is its ability to generate ultra-long reads, measuring >100kb up to currently several Mb (4). This capability allows full haplotype phasing, resolving complex genomic regions, and characterizing genomic variations that are challenging or even impossible to analyze with short-read sequencing technologies, e.g., centromeres, telomeres, or the 5-6Mb Major Histocompatibility complex region (3, 13, 14). Long-read sequencing has opened new possibilities for structural variant detection, full-length transcriptomics, the analysis of highly repetitive genomic regions, and end-to-end *de novo* assembly of pathogen and human genomes (15–17). However, as the major factor that determines read length is the quality of the input material, high nucleic acid integrity and purity are prerequisites for capturing ultra-long reads.

### 2.3 Accessibility

Compared to other sequencing platforms, ONT MinION, GridION, and PromethION devices are lightweight and have a small footprint, enabling bedside and field-based diagnostics. These portable devices facilitate real-time genomic analysis in remote settings, reduce reliance on centralized sequencing facilities and empower healthcare providers in resource-limited environments. The lower cost of ONT sequencing systems compared to current competitor platforms while maintaining the NGS advantage of sample barcoding and pooling to reduce costs, make this technology accessible for clinical and research groups with a lower budget. The ability to perform cost-effective, on-site sequencing has significant implications for infectious disease surveillance, outbreak response, and point-of-care diagnostics, enabling rapid decision-making and timely interventions.

Another strength of the ONT platform lies in its flexibility, allowing users to set up their own analysis pipelines and environments that build on ONT’s openly available base-calling and data processing tools, eliminating the dependence on a fully centralized compute infrastructure. Many ONT analysis workflows benefit from the use of Graphics Processing Units (GPUs), which are designed for high-throughput parallel processing. In ONT sequencing applications, GPUs are essential for real-time base-calling, raw signal processing, and large-scale sequence alignment. The performance of these tasks is directly influenced by the strength of the GPU: high-end GPUs enable faster and more complex analyses while modest GPUs, though slower, can still support many essential workflows. Many users, particularly researchers, configure their setups in-house by using computers with suitable capacities to handle ONT sequencing workloads. Fully customizing workflows for specific applications demands substantial computational know-how, which can be a powerful advantage for experienced users but can be a challenge for first-time-adopters or those with limited technical training.

Thus, despite the high accessibility of the ONT sequencing technology itself, ONT data analysis requires hands-off but sophisticated error-correction algorithms and application of hands-on bioinformatics tools, which increases the complexity of analysis, the demand for computational resources, and the capacity for data storage. One solution is offered through cloud-based computing or access to high performance compute clusters, which can help to accelerate analyses while providing version control and reducing costs (18). In addition, ONT is continuously developing user-friendly, containerized bioinformatics pipelines, providing a growing catalog of *Nextflow* workflows that are specifically tailored to ONT long-read data, e.g., for base-calling, assembly, and alignment, automated analysis of DNA methylation, telomeres (“*Telo-Seq*”), chromatin capture (“*wf-pore-c*”), RNA reads (“*wf-transcriptomes*”), single cell experiments (“*wf-single-cell*”), metagenomics and infectious disease typing (“*wf-metagenomics*”, “*wf-16s*”, “*wf-mpx*”, “*wf-flu*”, “*wf-tb-amr*”), as well as structural and single nucleotide variants (“*wf-human-variation*”, “*wf-pgx*”) (19). The use of containerization not only simplifies deployment but also promotes standardization of pipelines and reproducibility of results across different computational environments. In 2025, ONT and Cepheid announced a strategic collaboration to integrate GeneXpert cartridge-based sample preparation with ONT sequencing, aiming for a scalable, automated, “hours not days” infectious disease workflow, a clear move toward implementing more hospital- and laboratory-friendly diagnostics. This combination of openly available algorithms, customizable setups, *Nextflow* integration, and scalability in the cloud makes ONT sequencing one of the most accessible and adaptable platforms for research across a wide variety of settings and has great potential for clinical use, where version control and performance consistency are key.

### 2.4 Adaptive sampling

**Figure 2:**
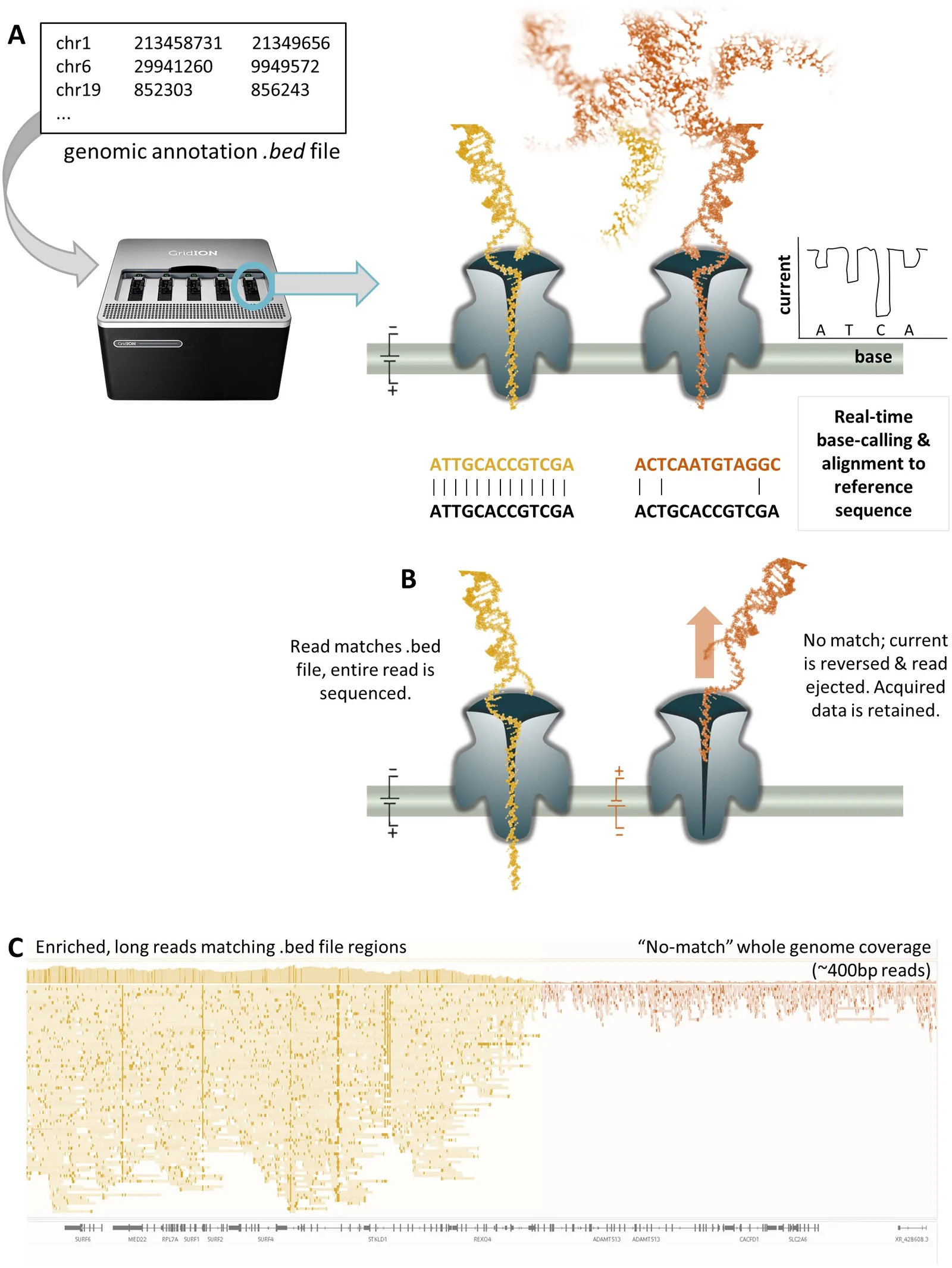
Adaptive sampling allows for real-time selective enrichment of specific genomic regions. **(A)** An annotation file listing all region of interest coordinates is provided to the ONT sequencer. The ionic current changes measured while each native nucleotide strand passes through a Nanopore is translated in real-time to base information (A,T,C,G, 5mC, 5hmC, 6mA, U, PseudoU, m6A and others) and mapped to the provided reference genome. **(B)** After sequencing the first ~400 bases, an algorithm checks whether the sequence matches a provided region of interest or not. In case of a match, the complete molecule is sequenced and a long read is produced. In case of a no-match, the current in this particular pore is reversed and the molecule ejected but the ~400bp long read mapping to an off-target region is retained. This process happens in parallel at thousands of pores at a time. **(C)** The result is an enrichment of genomic target regions that are captured with long reads (left) and a lower whole genome coverage with 400bp long rejected reads (right) that allows copy number and similar analyses.

*Adaptive sampling* enables the selective enrichment or depletion of specific genomic regions in real-time by providing the ONT sequencer with an annotation file in the common*.bed* format. Thus, the sequencing coverage for regions of interest is increased without the need for PCR-based pre-amplification or targeted library preparation (**Figure 2**). Multiple regions can be targeted simultaneously with or without surveillance of base modifications, allowing enrichment or depletion of up to 10% of the human genome. While increasing coverage of target regions, shorter (rejected) reads accumulate and allow to capture the complete genome at a reduced “background” coverage of 2 to 10x coverage, which can be useful for copy number variation (CNV)- and similar analyses. Another advantage is that *adaptive sampling* strategies can be paused and adjusted in real-time based on the captured data (20). Due to its versatility, simplicity of adding or removing analysis targets, reduction of not-of-interest genomic areas, and ability to resolve highly variable genomic regions, ONT *adaptive sampling* is a highly attractive approach for clinical genomics. Early studies have demonstrated it as a powerful tool for haplotype phasing, determining copy number variations as well as breakpoints and direction of structural variants, e.g., in X-chromosome inactivation quantification, rare genetic diseases, neurological repeat expansion diseases, and PGx studies (21–27). *Adaptive sampling* has also proven useful for enriching small supernumerary marker chromosomes and mitochondrial DNA (28, 29).

In pioneer clinical cancer settings, *adaptive sampling* has been reported to improve the characterization of large-scale genomic rearrangements, and to accurately detect structural- and single nucleotide variants in germline solid tumors, central nervous system tumors, and leukemias. In these studies, detection of genomic driver alterations, methylation classification, and DNA copy number information was possible in real-time in as little as 15-30min, followed by more comprehensive molecular profiling (e.g. complex CNV detection) within 6-24h and was possible even at low coverage (10x) (30–33). *Adaptive sampling* has also been successfully employed in fast and accurate antimicrobial resistance testing and in an *S. pneumoniae* surveillance strategy, where it was found to be feasible for low-resource settings, for novel phenotype and serotype discovery, and for rapid outbreak investigation (34–36).

Due to the novelty of *adaptive sampling*, some aspects currently impact on its suitability as a clinical test: Identifying and optimizing the highly flexible *adaptive sampling* run parameters (e.g., harmonizing read length, pore health, and*.bed* file configuration) requires some testing and computational expertise. Especially when barcoding and combining samples for sequencing, adaptive sampling can be very cost-effective as long as the number of samples and/or the number of regions of interest is high, but might become expensive if either or both are low. In addition, adaptive sampling for high confidence variant detection in human samples currently requires a large amount of nucleic acid input and is not yet suitable for high sensitivity approaches (37), although sensitivity issues could be overcome in some settings, e.g., in pathogen surveillance and microbiome studies (38–41). Adaptive sampling is still a new technique and advancements will likely overcome some or all of its current limitations, which are dwarfed by its most compelling advantages: PCR-free enrichment and a very fast total turnaround time of only 4 to 24h (33, 38, 42).

## 3 Application in transplantation medicine

### 3.1 Hematopoietic stem cell transplantation

The 3Mb long and highly polymorphic human leukocyte antigen (HLA) region harbors the 11 genes that are typed in order to identify a well-matched HSC donor for timely transplantation and optimized patient survival. The gold standard for selecting HSC donors requires high-resolution HLA typing at the allelic level for critical HLA loci, specifically *HLA-A, -B, -C, -DRB1, -DQB1* (and sometimes *-DRB3/4/5*, and -*DPB1*). Donor/recipient mismatches in these HLA alleles have significant impact on immunologic events increasing the risk of graft-versus-host disease (GvHD) and reducing recipient survival (43, 44). Although multiple studies have reported the impact of Killer Cell Immunoglobulin-like Receptor (KIR) and leukocyte immunoglobulin-like receptor (LILR) gene matching on transplantation success (45–47), neither is yet routinely performed in clinical practice due to non-uniform study results, the diversity and complexity of the genomic loci, a lack of references databases as well as access to long-read sequencing technology (48).

Depending on the clinical center, gold standards for sequencing-based typing use PCR pre-amplified sequences for 5–12 *HLA* genes, with obvious limitations being difficulties to phase these reads and an inability to detect large structural and novel variants. Due to its rapid turnaround and ultralong read capacity, ONT sequencing can overcome these limitations and therefore has the potential to revolutionize HLA typing and donor-matching in HSC transplantation as well as to improve risk assessment. In a proof-of-principle study in 33 clinical samples, Matern et al. compared ONT *vs*. Sanger sequencing in 70 HLA class I allele groups and demonstrated a 100% typing concordance between both techniques. In an independent validation cohort of 67 samples, they report that ONT sequencing showed complete concordance with reference methods and support its feasibility for routine histocompatibility testing (49). In addition to donor matching, ONT sequencing has also been validated for gut microbiome surveillance as well as for rapid diagnosis and treatment monitoring of serious infections in HSC transplantation contexts, which significantly reduced infection-related mortality (50–52).

### 3.2 Solid organ transplantation

To maintain viability of transplantable organs from deceased donors, they must be screened for infection and, if high-resolution HLA matching is desired, HLA typed within 4 to 12h, hence a rapid turnaround is critical. Typing in this context is increasingly performed in the form of epitope-based matching, enabling personalized donor-recipient compatibility beyond conventional HLA matching. This matching approach considers the specific amino acid sequences of HLA molecules, improving the accuracy of compatibility assessment and reducing the risk of rejection. As HLA genotyping for deceased donors is particularly time-sensitive, NGS and PacBio long-read sequencing have not seen widespread adoption in clinical laboratories for this purpose (48), whereas targeted ONT sequencing is already being applied. Both the Genome Canada Transplant Consortium and the Fiona Stanley Hospital in Perth, Australia, have demonstrated the feasibility of high-throughput epitope-based matching using ONT sequencing, paving the way for wider clinical implementation (53, 54). Other multicenter proof-of-principle studies in Europe (55) and the US (56) reported ONT sequencing for HLA typing as a ‘laboratory in a suitcase’ assay that only required 40-50ng genomic DNA input with a total turnaround of 8h (with the time limiting factor being long-range PCR reactions taking up to 3 hours) and an assay cost of only ~$80 USD/sample. Further reports have demonstrated that ONT allows identification of novel HLA subtypes (57–60) and outperforms microbial cultures in terms of detection speed, sensitivity, and identification of rare pathogens in deceased donors and transplant recipients (61, 62). In a proof-of-concept study, Montgomery et al. have shown that calling HLA genotypes simultaneous to quantifying HLA class I gene expression is possible and clinically informative when employing ONT sequencing on mRNA molecules (63).

### 3.3 Pharmacogenomics and drug-related adverse reactions

Beyond transplantation and donor-recipient matching applications, ONT sequencing can also support pharmacogenomics (PGx) by identifying clinically actionable variants associated with drug efficacy and adverse drug reactions. Drug response information, including carriage of specific HLA-B* alleles, e.g., HLA-B*57:01 (abacavir hypersensitivity) or HLA-B*15:02 (carbamazepine hypersensitivity) (64), enables personalized prescribing practices, minimizes the risk of adverse drug reactions, and improves patient safety. Integration of PGx loci into other sequencing-based tests, and eventually into electronic health records could support real-time clinical decision-support and empower healthcare providers with actionable genetic information for return of results at the point of care. The simplicity of incorporating hundreds of relevant PGx loci to an existing panel of 50 Mendelian regions of interest has recently been demonstrated in an *adaptive sampling* study, which focused on the diagnosis of tandem repeat expansion disorders (25). So far, a variety of targeted ONT sequencing powered PGx studies have reported in-depth characterizations and haplotype-resolving protocols for drug and disease-focused multiplex panels (65, 66) as well as for very large or complex PGx genes such as *DPYD* (67), *CYP2D6* (68), and *SLC28A3* (69). In a systematic approach, Deserranno et al. utilized ONT *adaptive sampling* to target 1,036 PGx-relevant genes and demonstrated high accuracy in variant and haplotype detection that allowed for sample multiplexing without loss of performance. The authors note that the greatest limitation of this methodology is the current lack of bioinformatics tools to are able to accurately call star alleles from ONT data (70). A 2025 reanalysis of this data using ONT’s latest base-, variant-calling and phasing tools achieved correct *CYP2D6* star-alleles and superior variant phasing (≈3× more variants per phasing block) for all Clinical Pharmacogenetics Implementation Consortium (CPIC) Level A genes compared to the Twist long-read PGx panel (71). As ONT protocols allow for direct, full-length RNA sequencing, approaches similar to Montgomery et al.’s study of HLA transcripts (63) could also be applied as valuable strategies for pharmacogenomic assessments, allowing the identification of genetic variants from RNA sequences while assessing drug responses at the level of target gene activation and -expression or machine-learning assisted prediction of such responses. Regardless of the type of nucleotides sequenced, to be successful, comprehensive disease susceptibility and drug response analyses require a full representation of genetic diversity in the utilized reference genomes. Although longer and more accurate reads and references have improved our ability to identify ancestry-linked and rare genetic variants (72), our diagnostic capabilities remain limited in fully resolving PGx- and other medically relevant genes as long as our reference genomes do not adequately reflect all global populations.

### 3.4 Emerging immunology applications beyond PGx and HLA typing

While HLA typing represents one of the most mature immunological applications of ONT sequencing, the technology has potential to expand genomic characterization across a broad range of immune loci. Many clinically relevant immune genes remain difficult to resolve using short-read sequencing due to extensive polymorphism, copy-number variation, and structural complexity. Long-read ONT sequencing increasingly enables comprehensive characterization of these challenging regions, including *KIR*, *LILR*, and Fc gamma receptor (*FCGR*) loci. Recent studies have demonstrated accurate haplotype-resolved *KIR* and *ABO* sequencing (73–75), and emerging applications (76) suggest that ONT sequencing may enable haplotype-resolved characterization of the *FCGR* locus together with assessment of structural variation and regulatory features that have historically been difficult to interrogate using short-read approaches. Such high-resolution characterizations may improve our understanding of NK-cell biology, antibody-mediated immunity, immunotherapy responses, and transplant outcomes.

Beyond genomic variation, ONT sequencing enables direct analysis of full-length RNA molecules and native DNA modifications. These capabilities create opportunities for profiling immune-cell transcriptomics (77), integrated genetic and epigenetic profiling (75), as well as T-cell receptor (TCR) and B-cell receptor (BCR) repertoire characterization. Simultaneous assessment of gene expression, alternative splicing, and DNA methylation may provide novel insights into immune activation, tolerance, immune aging, and autoimmune disease pathogenesis.

In transplantation, future applications may extend beyond donor-recipient HLA matching toward comprehensive alloimmune risk assessment. Integration of *HLA*, with blood group and immune function-associated gene sequences, PGx, epigenetic, and potentially transcriptomic information could support improved prediction of rejection risk, immune-mediated complications, and responses to immunosuppressive therapies. As analytical methods mature, nanopore sequencing may evolve from a high-resolution typing platform into a broader precision-immunology framework capable of supporting disease risk prediction, longitudinal immune monitoring, and personalized clinical decision-making.

## 4 Application in infectious disease management

### 4.1 Rapid pathogen identification and personalized antimicrobial therapy

In 2019, total annual deaths from infections were estimated at 13.7 million worldwide, of which 1.27 million were directly caused by antimicrobial resistance (AMR) and in an additional 3.6 million of these deaths, AMR played a contributing role (78). Rapid detection of pathogen sequences allows for the identification and monitoring of host adaptation, diagnostic targets, response to vaccines, and pathogen evolution (79). Our ability to rapidly identify resistance genes, virulence factors, and mobile genetic elements in bacteria to predict resistance phenotypes is crucial for preventing the spread of drug-resistant infections and preserving the effectiveness of antibiotics. If combined with clinical prediction rules and genomic data, observations from comprehensive studies such as the Study for Monitoring Antimicrobial Resistance Trends (SMART) (80) will facilitate expert antimicrobial stewardship applications and guide treatment policies to optimize patient care.

ONT sequencing has not only advanced the discovery of AMR genes in non-human and human pathogens such as *P. aeruginosa* (81, 82), *N. gonorrhoeae* (83), *Campylobacter* (84, 85), *K. pneumoniae* (86), and *M. tuberculosis* (87–90), but has also been extensively employed for retrospective metagenomic analyses of mono- and polymicrobial infections, enabling rapid adjustments in antimicrobial therapy and improved outcomes in complex infectious diseases (91–94). Recent studies comparing the accuracy of ONT to slower sequencing methods, mass spectrometry, and culture-based assays demonstrated comparable to superior performance of ONT protocols in AMR gene detection as well as equivalent virulence factor identification, achieving reproducibility accuracies of 98 to 100% (90, 95–99). Whereas culture-based methods predominantly support growth of Gram-positive bacteria, direct ONT sequencing allows for the unbiased identification of Gram-negative species (100), and can accurately detect low-abundance plasmid-mediated resistance, which often remains undetected by conventional diagnostics (101).

Moving away from culture-based assays together with the integration of sequence enrichment methods and advanced machine learning into diagnostic ONT workflows have already revolutionized the field (102–106). Retrospective applications of ONT sequencing for diagnosis and treatment of infectious diseases have been recently reviewed in respiratory tract (107–109) and in fungal infections (110). Moreover, ONT has developed “NO-MISS” extraction protocols to guide users in optimal isolation of different pathogen species (111) and additional ONT experimental protocols and analytical strategies for pathogen detection have been collected and compared (112–114). The growing ONT-based AMR research community has provided sophisticated bioinformatics tools and automated, containerized pipelines for user-friendly metagenomic long-read analysis, e.g., for taxonomic assignment, AMR gene identification, AMR origin prediction, real-time plasmid transmission detection, and community analysis (115–117).

Several proof-of-concept prospective clinical studies have been conducted and reported high accuracy of ONT sequencing in pathogen and AMR gene detection that is superior to culture-based approaches in as low as 4h turnaround time, e.g., in patients with suspected bloodstream infections (118–120), wound infections (121), and drug-resistant tuberculosis infections (90). Moreover, ONT sequencing has proven highly useful for targeted viral diagnostics, antiviral resistance detection and -monitoring (122–128). A challenge for sequencing-based approaches in this field remains the simultaneous performance of RNA- and DNA-based diagnostics which require different workflows. Although RNA-to-DNA conversion is possible, current protocols introduce base errors, bias, and irreversibly strip methylation information.

In addition to pathogen identification, long-read sequencing strategies also enable characterization of host-pathogen interactions (129), immune evasion mechanisms (130), and antigenic variation (131).

### 4.2 Epidemiology, public health and outbreak surveillance

Nanopore sequencing provides all the necessary capabilities for real-time outbreak investigation, enabling the identification of transmission pathways and implementation of effective public health measures. In large-scale retrospective proof-of-concept surveillance studies, ONT approaches have already been deemed suitable for infection tracing and outbreak analysis of multidrug-resistant organisms in hospital environments such as in neonatal intensive care units (132–134), regional (135) and cross-border pathogen tracing approaches (136) albeit with varying performance for *P. aeruginosa* (137). ONT sequencing was also successfully employed to confirm a laboratory cross-contamination that led to false positive diagnosis of a series of *M. tuberculosis* infections in Canada (138). In a prospective field evaluation to test targeted ONT sequencing against the gold standard for tuberculosis drug resistance prediction in Zambia and South Africa, Schwab et al. reported variable ONT sequencing success rates that they found to be proportional to the bacterial load of a sample. Despite faster diagnostic results (days) compared to the gold standard (~2 months) and high specificity, the ONT test sensitivity varied by variant (33-94%), indicating that technical improvements are required before broader implementation (139), which were addressed in an independent multicenter study (140) and led to the 2024 WHO approval of the ONT *AmPORE TB* assay. WHO endorsement of this rapid WGS-based alternative to culture-based drug susceptibility testing suggests a growing regulatory confidence in nanopore sequencing technology for high-stakes infectious disease decisions. Preliminary data further suggests that integrating ONT sequencing into *P. falciparum* genomic surveillance could lead to significant advancements in malaria control (141).

Large-scale retrospective applications of ONT protocols in viral outbreak tracing have thus far focused on both DNA viruses such as MPXV (Mpox) (142), ZIKV (Zika virus) (143), and Sars-CoV2 (144, 145) and RNA viruses such as *DENV* (Dengue virus) (146). ONT-based protocols have also been successfully employed in viral surveillance of water and air samples to anticipate human health risks (147, 148).

## 5 Other clinical and research applications

### 5.1 Oncology and liquid biopsy approaches

Nanopore sequencing facilitates non-invasive tumor monitoring through circulating blood and cell-free DNA (cfDNA) analysis, enabling early cancer detection and personalized treatment strategies. The ability to detect tumor-derived DNA in the bloodstream has significant implications for cancer screening, monitoring treatment response, and identifying minimal residual disease (MRD) as well as emerging resistance mutations (149–152). Integration of nanopore sequencing with multi-omics approaches can further enhance personalized oncology treatment strategies, providing a comprehensive view of tumor biology and guiding precision medicine. An example is the Personalized OncoGenomics program (POG), a precision medicine initiative at BC Cancer, Canada, that uses prospective genomic analysis to tailor treatments for advanced stage and metastatic cancer patients. POG reported that ONT-based sequencing significantly improves the validation, resolution, and classification of germline structural variants in enrolled patients with suspected or NGS-determined variants (153).

Similarly, the UNICANCER center in Dijon, France, reported an ONT *adaptive sampling* strategy that is suitable for the analysis of germline alterations in whole blood and effective at a 10x minimum coverage (31). Apart from whole genome and *adaptive* approaches, ONT sequencing of whole blood samples has successfully supported the development of targeted sequencing panels, mainly for hematologic malignancies. Such have been validated for rapid gene mutation detection in acute myeloid (AML) (154, 155) and chronic lymphocytic leukemia (CML) (156, 157) as well as for in-depth characterization of complex mutations and duplications as are, e.g., present in the *FLT3* oncogene (158). In a case report, ONT sequencing of a patient’s cerebrospinal fluid could resolve a 3-months clinical course without diagnosis to the diagnosis of a rare intravascular B-cell lymphoma, which directly translated to a therapeutic strategy and rapid clinical improvement (159). ONT sequencing also facilitated evaluation of immunoglobulin heavy variable gene mutations in chronic lymphocytic (CLL) and acute lymphoblastic leukemia (ALL), where it outperformed the NGS gold standard in terms of accuracy, simplicity of analysis, cost, turnaround time, and sensitivity, down to MRD detection levels (160, 161).

ONT sequencing was also successfully employed for MRD detection in a CML context - although not used as a longitudinal test itself, the technology enabled the precise definition of personalized genomic translocation junctions, e.g., in *BCR-ABL1* fusions, for cost-efficient PCR-based monitoring (162). Aside from genomics approaches, clinically actionable diagnostic classifications for acute leukemia samples could be derived from ONT mRNA sequencing data (163). Considering the rise in research development and clinical use of immunotherapies, ONT sequencing has been proven useful in determining polyclonal vector integration sites in clinical T-cell and other vector-based immunotherapies as well as in detecting *FCGR3A* gene polymorphisms that affect the efficacy of antibody-therapies (164, 165).

Also in cell-free samples from solid cancer patients, multiple studies have reported ONT sequencing to be highly suitable for clinical surveillance, e.g., in brain (166, 167) and breast cancer (168), for gastric cancer detection (169), mutant *TP53* profiling in head and neck cancers (170), detection of *EGFR* mutations in non-small cell lung cancer (NSCLC) (171), and monitoring of COSMIC mutational signatures in various solid cancers (172). Due to the size, range and complexity of structural variations, reliable detection of chromosomal or genomic rearrangements, indels, insertions, and copy number changes are more challenging in extracellular fluids but could be effectively detected using ONT sequencing, e.g., *EGFR*, *MYC, RICTOR* and *PIK3CA* amplifications in lung cancer (173), and gain of Chr.1q or loss of Chr.6q in brain tumors (174).

Another key determinant in liquid biopsy analysis is the absolute or relative proportion of circulating tumor DNA (ctDNA) within the total cell-free DNA, which could be effectively quantified using ONT-based techniques (170, 175, 176). Additionally, two of ONT’s unique characteristics, the simultaneous profiling of modified bases on native DNA and the generation of ultra-long reads, have been shown to be of value for ctDNA profiling as these allow to determine cell type and cancer-specific methylation changes as well as cancer-associated fragmentation signatures, which could be valuable for biomarker discovery (175, 177–179).

Although so far only demonstrated in pioneer studies, ONT-seq has great potential to improve characterization of immunotherapy biomarkers, antigen presentation pathways, HLA loss of heterozygosity (180), neoantigen generation (181), Fc receptor polymorphisms (182), and immune-cell therapy engineering (183).

### 5.2 Intra-surgical diagnostics

Intraoperative diagnostics can support personalized medicine by changing surgical management, e.g., by outlining tumor margins or aiding real-time tumor classification, but such assays need to be ultra-rapid, easy-to-use, and ideally automatable. In a proof-of-principle study, Wadden et al. were able to achieve 250x sequencing coverage and called a known *H3F3A* histone variant from a pediatric tumor sample within intra-operative time frames of <35 min with an estimated cost of ~$388 USD/sample (184). In a simulated intraoperative setting, the ONT-based *MethyLYZR* approach achieved highly accurate molecular classification of 10 biopsies from nervous system malignancies with 100% concordance and a total turnaround time of 40 min (185). Using a similar ONT sparse methylation profiling approach, Vermeulen et al. prospectively demonstrated their workflows’ applicability during 25 surgeries, achieving a diagnostic turnaround time of less than 90 min. Of these, 18 (72%) diagnoses were correct and 7 did not reach the required confidence threshold (186). These promising pilot studies pave the way for the development of other rapid ONT-based diagnostic protocols, which could help to reduce the need for repeat surgeries and inadvertent injury to healthy tissues and could enable tailored surgical procedures or other intra-operative treatments.

### 5.3 Complex genetics and rare disease diagnostics

Rare diseases comprise complement disorders, autoinflammatory syndromes, primary immunodeficiencies, HLA-associated disease risk, and diseases with immune dysregulation components. Their diagnosis requires serial testing and can take years due to several healthcare system barriers. Even in expert clinical settings applying NGS-based methodologies, every second patient currently remains undiagnosed (187). ONT sequencing has the potential to consolidate stepwise testing into a single assay, enabling rapid detection of structural variants and exact chromosomal breakpoints in complex genetic disorders which short-read NGS or hybridization approaches cannot resolve. This has been demonstrated via ONT sequencing for e.g., complement factor H rearrangements (188), causal variants of Carney complex (189), Gaucher disease (190), spastic-ataxia (191), muscular dystrophy (192), and antithrombin deficiency (193). In a number of proof-of-principle studies, DNA methylation-aware ONT sequencing has been used to advance clinical genomics workflows for rare disease detection (21, 194–202), including an *adaptive sampling* strategy to simultaneously profile 37 genes in which short tandem repeat expansions can cause over 50 neurological and neuromuscular diseases (26). Such methylation sensitive approaches enable the real-time diagnosis of imprinting disorders and methylation dependent rare diseases, and can therefore facilitate timely intervention.

This is of particular importance in neonatal and pediatric intensive care units (ICUs), where rapid diagnosis can significantly improve outcomes (203). Testing a rapid whole genome ONT sequencing platform in critically ill children with genetic diseases, Clark et al., were able to correctly diagnose 3/7 ICU infants in under 16 hours (204) and Kamolvisit et al. reported a 61% diagnostic rate (11/18) with a median turnaround of 9 days (205). Owen et al. could infer a clinical diagnosis of thiamine metabolism dysfunction syndrome 2 within 17 hours for a 5-week-old patient (206). Further, the New Zealand Newborn Genomics Programme reported an acute care clinical ONT workflow that is able to provide rapid precision medicine for critically sick children on a national scale. The group reported that they could accurately detect copy number variation at 2x and small variants at 40x coverage, with additional sequencing depth not improving the accuracy and sensitivity (207). Similarly, ONT sequencing can facilitate preimplantation diagnosis (208, 209) as well as diagnosis of fetal aneuploidies and birth defects in under 4 hours (210, 211).

In addition to the fast turnaround, ONT sequencing enables researchers and clinicians to explore pathogenetic variants outside the coding elements of a gene, e.g., in regulatory elements and splice sites, which could account for some of the ~50% of suspected Mendelian conditions that cannot be resolved with current clinical testing approaches. Using ONT sequencing, Viering et al. could identify additional intronic pathogenic variants in the *SLC12A3* gene in 45/67 patients which they could associate with symptom severity of Gitelman syndrome (212). Furthermore, ONT sequencing allows for the precise identification of transposable element insertion and deletion sites which were suggested to be causative for genetic disorders such as antithrombin deficiency (193, 213) and has been successfully applied in identifying structural alterations in mitochondria, which cause primary mitochondrial diseases that can lead to multi-organ failure (214). Another successful application of ONT sequencing in this context is the identification of rare blood groups to prevent hemolytic disease and transfusion reactions (215).

## 6 Future directions and challenges

### 6.1 Standardization, centralization, and version control

Nanopore sequencing is emerging as a transformative tool in precision medicine, offering rapid, portable, native, and long-read sequencing capabilities at high data quality. Experimental steps have been taken to apply the ONT platform to protein analysis and sequencing, to single-molecule covalent chemistry, and clinical sensing applications (216, 217). In theory, a single nanopore sequencing run could replace many of the multi-step clinical tests we use today, saving patient wait time and healthcare resources. However, for one-step, centralized genomic testing to become reality, several administrative factors would need to be addressed on multiple levels. Departments, hospitals and health authorities would be required to work together to streamline diagnostics, staff training, and financial aspects of testing.

Further, concerns about privacy, storage, and ownership of the generated data would need to be addressed, as well as how to handle the reporting of “incidental findings”, which might require centralized pre- and post-testing counselling to be made available to patients. Standardization of analysis workflows will be essential for regulatory approval and to ensure the reliability and reproducibility of nanopore sequencing based clinical applications. Although many task-specific ONT long-read data analysis pipelines and tools have been developed for and by researchers, most of these are not yet suitable for clinical diagnostics, as they may lack user-friendliness, integrated confidence metrics, dedicated updates and support, and robust version control (218). Performance can also vary widely between tools, adding further variability to analysis outcomes. ONT-developed software evolves rapidly to best support cutting-edge research, requiring frequent updates and computational expertise on the user’s side. ONT has announced to address requirements for clinical standardization and version control in form of a “Q-Line” for applications-focused users ensuring greater consistency, reliability, and regulatory readiness (219). How Roche SBX analytical workflows will look like has not yet been announced by the company.

### 6.2 Technical and resource limitations

Despite great improvements in chemistry and computational post-sequencing correction (220–222), ONT long read sequencing still faces several technical limitations when compared with other sequencing technologies, including lower per-read accuracy, reduced sequencing yield, challenges with homopolymer regions (especially those longer than 10 bases), as well as biases and challenges in data processing, computational demand, and cost.

To perform high resolution HLA genotyping, El-Lagta et al. compared the newest (R10.4.1) vs older ONT flow cell chemistries against Ion Torrent sequencing performance. In this comparison – without application of duplex sequencing – R10.4.1 demonstrated significant signal-to-noise reduction and an increase in median Phred quality (19.7) and raw read accuracy (97.6 - 97.9%), which although being a great improvement over the legacy R10.3 ONT flow cell chemistry, was still lower than the accuracy achieved with the Ion Torrent platform (99.2 – 99.3%, using BLAST or LASTAL alignments, respectively). When performing targeted HLA typing of 924 samples, the authors described a 1.8% failure rate due to insufficient read depth across one or more HLA loci, which could be resolved by increasing the data yield to above 0.175 Gb (223). In general, R10.4.1 sequencing yield appears to be lower compared to previous flow cell models (R9.4.1) due to slower DNA translocation through the pores (224). This can impact coverage and necessitate higher DNA input and/or longer sequencing times for comparable data output, particularly in metagenomic or large genome projects. Although duplex sequencing can dramatically increase per-read accuracy, experimentally realized duplex read yields were reported to be lower than the manufacturer claims, meaning most reads are still single-stranded and less accurate (224). Current error rates for difficult templates (e.g., high-identity 16S rRNA sequencing or high-GC genomes) may not meet all taxonomists’ or clinical standards and generally require post-processing (222). Another limitation of ONT flow cells originates from the use of biological nanopores as there is substantial flow-cell-to-flow-cell variability in the availability and activity of pores, which directly translates to sequencing output (223).

Cost-effectiveness considerations are crucial for facilitating widespread clinical implementation of a test, particularly in resource-limited settings, to ensure responsible and equitable access to nanopore sequencing technology. Currently, less than 20% of low- and middle-income countries have access to modern data infrastructure (225), removing the possibility of cloud computing in many places and introducing a substantial access bias. Further, despite pioneer efforts in building references catalogues from long-read sequencing (226–229), the current lack of reference data across species and nucleotide or base modification subtypes, remains one of the biggest barriers in using ONT sequencing data to its full potential, e.g., for filtering structural variants or for integration of artificial intelligence (AI) for biomarker discovery and clinical data interpretation.

### 6.3 Toward distributed genomic medicine

We believe that the long-term impact of nanopore sequencing may extend beyond technical improvements in genomic testing to fundamentally reshape how precision medicine is delivered. In the near term, portable sequencing platforms may enable genomic testing to be performed in regional hospitals and transplant centers, while cloud-based pipelines and centralized expert interpretation support same-day reporting of clinically actionable results such as high-resolution HLA typings. Over the next decade, genomic testing is likely to become increasingly integrated into routine clinical care, with sequencing triggered automatically by defined clinical events and linked to electronic health record decision-support systems that are capable of delivering automated pharmacogenomic and risk-based recommendations. Ultimately, a single comprehensive genomic assay could support multiple applications throughout an individual’s lifetime, including transplantation, pharmacogenomics, disease diagnosis, and infectious disease surveillance, reducing the need for multiple independent testing platforms. In this model, sample collection and sequencing become increasingly decentralized and patient-facing, allowing clinicians and laboratories to act rapidly, whereas computational infrastructure, data governance, algorithm- or AI development, and clinical interpretation remain coordinated through provincial or national genomic networks. In many respects, this model reflects the ongoing development of large-scale learning health systems and genomic medicine infrastructures, including existing initiatives such as GEMINI in Canada and national genomic medicine programs in the United Kingdom and United States, which increasingly combine distributed clinical data generation with centralized data governance, analytics, and knowledge generation (230–232).

Such a model also mirrors the evolution of other specialized laboratory services, in which testing is performed across multiple regional centers within a shared quality-management framework. Nanopore sequencing-enabled testing could be similarly distributed across major hospitals and independent programs, while bioinformatic analysis, knowledge management, and advanced data interpretation are consolidated within centralized provincial or national hubs. As sequencing turnaround times continue to decrease, the principal bottlenecks are likely to shift from data generation toward interpretation, clinical reporting, and electronic health record integration. Bioinformaticians, data scientists, and software engineers may therefore become integral members of clinical laboratory teams, supported by automated pipelines, AI-assisted variant prioritization, clinical decision-support systems, and standardized reporting frameworks. Such networked models could facilitate rapid deployment of genomic testing while maintaining consistency, regulatory oversight, and equitable access to specialized expertise across geographically distributed healthcare systems.

## 7 Conclusion

Nanopore sequencing has emerged as a transformative tool in precision medicine, offering rapid, flexible, and portable long-read sequencing capabilities. Long reads being captured directly from native DNA circumvent the limitations of short-read technologies while adding epigenomic and epitranscriptomic information, bringing us one step closer to interpreting (patho)physiological states in tissues and cells. Targeted ONT sequencing is being widely used for rare variant detection, structural variant analysis, and full haplotype assignment, which are essential for personalized drug dosing and adverse effect risk prediction in various disease contexts. *Adaptive sampling*, a PCR-free, on-device enrichment of target loci that can be quickly adapted to newly defined targets, has recently entered the clinics, demonstrating improved resolution of complex alleles and tremendous flexibility. Pioneer long-read sequencing applications span transplantation, infectious disease management, oncology-, pharmacogenomics-, and rare disease diagnostics. Recent advances now point toward a converging ecosystem in which ultra-rapid whole-genome sequencing, increasingly mature PGx-focused *adaptive sampling* workflows, and emerging automated infectious-disease pipelines collectively enable true sample-to-answer genomics within hours. The integration of 24-hour WGS protocols with haplotype-resolved PGx panels and ONTs development of cartridge-based, near-automated pathogen sequencing platforms illustrate how multi-layered outputs (germline, PGx, AMR, structural variants, and epigenetics) can be generated in decentralized, non-specialist laboratories. Together, these developments signal a transition from promising prototypes to scalable, clinically deployable precision-medicine workflows. As these workflows and technologies continue to evolve, their integration into routine clinical practice promises to enhance patient care, to provide broad access to rapid, actionable molecular insights, and to expand the frontiers of -omics medicine. The realization of real-time diagnostics and personalized medicine is within reach, paving the way for a new era of healthcare driven by rapid and comprehensive -omics insights.

### 7.1 Search strategy and selection criteria

Relevant literature was identified using PubMed, searching for combinations of the terms “Nanopore sequencing” OR “Adaptive Sampling” with either of the following: “Transplantation”, “Pharmacogenomic”, “Pathogen”, “Antimicrobial resistance”, “Epidemiology”, “Diagnostic”, “Diagnosis”, “Oncology”, “Biopsy”, “Genetic Testing” and “Rare disease”. All articles that had a clinical focus were considered and included as long as the abstract was available in English and the used methodology was clearly identified as Nanopore sequencing. Additional references were identified through manual review of citations within the selected articles.

## The Genome Canada Transplant Consortium

Canada: Paul A. Keown^1,4^, Karen R Sherwood^4^, Franz Fenninger^4^, Jenny Tran^4^, Sarah Grasedieck^1^, James Lan^1,4^, Lenka L. Allan^4^, Bruce McManus^4^, Günther OP^5^, Ruth Sapir-Pichhadze^6^, Stirling Bryan^7^, Timothy Caulfield^8^, Ioannis Ragoussis^9^, Sarah Reiling^9^, Karim Oualkacha^10^, Kathryn Tinckam^11,12^, Robert Liwski^13^, Anna Greenshields^13^, Patricia Campbell^14^, Heloise Cardinal^15^, Sacha A. De Serres^16^, Chee Loong Saw^17^, Claude Daniel^17^, Michael Mengel^18^, Banu Sis^18^, Eric Wagner^19^, Noureddine Berka^20^, W. Robert McMaster^21^, Marie-Josée Hebert^22^, Leonard J. Foster^23^, Fabio Rossi^24^, Christoph H. Borchers^25^, Ciriaco A. Piccirillo^26,27^, Constantin Polychronakos^28^, Raymond Ng^29^, Anthony Jevnikar^30^, Pieter Cullis^23^, Guido Filler^31^, Harvey Wong^32^, Bethany Foster^33^, John Gill^1^, S. Joseph Kim^11,12^, Lee Anne Tibbles^33^, Atul Humar^11^, Steven Shechter^34^, Prosanto Chaudhury^35^, Nicolas Fernandez^36^, Elizabeth Fowler^37^, Bryce Kiberd^38^, Jagbir Gill^39^, Marie-Chantal Fortin^15^, Scott Klarenbach^40^, Robert Balshaw^41^, Seema Mital^42^, Istvan Mucsi^11,12,43^, David Ostrow^1^, Calvin Stiller^44^, Rulan S. Parekh^11,12,42^, Lucie Richard^45^, Lynne Senecal^46^, Tom Blydt-Hansen^47^, Matthew Kadatz ^4,39^, Anat Fisher^1^, Maziar Riazy^4^, Mike Barnett^1^, Anne Halpin^48,49^, Esme Dijke^48,49^, Jeffrey Kiernan^50^, Jiangping Li^51^, Marie-Christine Meunier^52^, Ahmed Mostafa^53^, Graeme Quest^54^, Christine Ribic^55^, Abubaker M E Sidahmed^56^ & Chee Loong Saw^57^. United States: Rene Duquesnoy^58^, Henry Erlich^59^, Howard Gebel^60^, Eric Weimer^61^, Bruce Kaplan^62^ & Gilbert Burckart^63^. The Netherlands: Marcel Tilanus^64^ & Teun van Gelder^65^. Germany: Gerhard Opelz^66^ & Michael Oellerich^67^. France: Pierre Marquet^68^. Hungary: Zoltán Kaló^69^. Australia: Phil O’Connell^70^, Jennifer Li^71^, Natasha Rogers^71^, Harry Robertson^72^ & Rob Carroll^73^.

^1^Department of Medicine, University of British Columbia, Canada

^4^Department of Pathology and Laboratory Medicine, Faculty of Medicine, University of British Columbia, Vancouver, British Columbia, Canada

^5^Günther Analytics, Vancouver, Canada

^6^Department of Medicine, McGill University and MU-HRI, Montreal, QC, Canada

^7^School of Population and Public Health, University of British Columbia, Vancouver, Canada

^8^Faculty of Law and School of Public Health, University of Alberta, Edmonton, Canada

^9^Department of Human Genetics, McGill University, Montreal, Canada

^10^Department of Mathematics, University du Quebec à Montreal, Montreal, Canada

^11^Department of Medicine, University of Toronto, Toronto, Canada

^12^Division of Nephrology, Toronto General Hospital, University Health Network and University of Toronto, Toronto, Canada

^13^Department of Pathology, Dalhousie University, Halifax, Canada

^14^Department of Medicine and Dentistry, University of Alberta, Edmonton, Canada

^15^Department of Medicine, University of Montreal, Montreal, Canada

^16^Department of Medicine, Laval University, Quebec City, Canada

^17^HLA Laboratory, Hematology Division, McGill University Health Centre, Montreal, Canada

^18^Department of Laboratory Medicine & Pathology, University of Alberta, Edmonton, Canada

^19^Department of Microbiology, Infectious Diseases, and Immunology, Laval University, Quebec City, Canada

^20^Department of Pathology, University of Calgary, Calgary, Canada

^21^Department of Medical Genetics, University of British Columbia, Vancouver, BC, Canada

^22^Research Centre, Université de Montréal, Montreal, Canada

^23^Department of Biochemistry and Molecular Biology, University of British Columbia, Vancouver, Canada

^24^School of Biomedical Engineering, University of British Columbia, Vancouver, Canada

^25^Department of Oncology, McGill University, Montreal, Canada

^26^Department of Microbiology and Immunology, McGill University, Montreal, Canada

^27^Research Institute of McGill Health Center, Montreal, Canada

^28^Department of Pediatrics, McGill University, Quebec, Canada

^29^Department of Computer Science, University of British Columbia, Vancouver, Canada

^30^Department of Microbiology and Immunology, Western University, London, Canada

^31^Department of Pediatrics, Western University, London, Canada

^32^Faculty of Pharmaceutical Sciences, University of British Columbia, Vancouver, Canada

^33^Cumming School of Medicine, Division of Nephrology, University of Calgary, Calgary, Canada

^34^Sauder School of Business, University of British Columbia, Vancouver, Canada

^35^McGill University Health Centre & Research Institute, Montreal, Canada

^36^Université de Montréal, Montreal, Canada

^37^Kidney Foundation of Canada, Burnaby, Canada

^38^Division of Nephrology, Dalhousie University, Halifax, Canada

^39^Division of Nephrology, University of British Columbia, Vancouver, Canada

^40^Department of Medicine, University of Alberta, Edmonton, Canada

^41^George and Fay Yee Centre for Healthcare Innovation, University of Manitoba, Winnipeg, Canada

^42^Department of Pediatrics, The Hospital for Sick Children, University of Toronto, Toronto, Canada

^43^Kidney Transplant Program, Ajmera Transplant Centre, University Health Network, Toronto, Canada

^44^Department of Medicine, Western University, London, Canada

^45^Héma-Québec, Quebec, Canada

^46^Division of Nephrology, University of Montreal, Montreal, Canada

^47^Department of Pediatrics, University of British Columbia, Vancouver, Canada

^48^Faculty of Medicine & Dentistry, Laboratory Medicine & Pathology Department, University of Alberta, Edmonton, Canada

^49^Departments of Pediatrics, Surgery, and Immunology, University of Alberta, Edmonton, Canada

^50^Department of Laboratory Medicine & Pathobiology, University of Toronto, Toronto, Canada

^51^The Ottowa Hospital, Ottawa, Canada

^52^Cellular Therapy & Transplantation Program, Hopital Maisonneuve-Rosemont, Universite de Montreal, Montreal, Quebec, Canada

^53^USask College of Medicine, Saskatoon, Canada

^54^Pathology and Molecular Medicine, Queen’s University, Kingston, Canada

^55^Division of Nephrology, Department of Medicine, McMaster University, Hamilton, Canada

^56^Immunogenetics and Transplantation Laboratory, London Health Sciences Centre and Pathology and Laboratory Medicine, University Hospital London, London, Canada

^57^Division of Hematology, Royal Victoria Hospital, McGill University, Quebec, Canada

^58^Thomas E. Starzl Transplantation Institute, University of Pittsburgh, Pittsburgh, United States

^59^Children’s Hospital Oakland Research Institute, Oakland, United States

^60^Department of Pathology and Laboratory Medicine, Emory University, Atlanta, United States

^61^Department of Pathology and Laboratory Medicine, University of North Carolina, Chapel Hill, United States

^62^Department of Medicine, Baylor Scott and White Health Systems, Temple, United States

^63^Office of Clinical Pharmacology, US Food and Drug Administration, Silver Spring, United States ^64^Transplantation Immunology, Maastricht University Medical Center, Maastricht, Netherlands

^65^Department of Clinical Pharmacy and Toxicology, Leiden University Medical Center, Leiden, Netherlands

^66^Transplantation Immunology, University Hospital Heidelberg, Heidelberg, Germany

^67^Department of Clinical Pharmacology, University Medical Center Gottingen, Gottingen, Germany

^68^Pharmacology and Transplantation, INSERM, University of Limoges, Limoges, France

^69^Center for Health Technology Assessment, Semmelweis University, Budapest, Hungary

^70^Faculty of Medicine and Health and The Westmead Institute for Medical Research, The University of Sydney, Westmead, Australia

^71^Department of Renal Medicine and Centre for Transplant and Renal Research, Westmead Institute for Medical Research, Westmead Hospital, Westmead, Australia

^72^School of Mathematics and Statistics, The University of Sydney, Sydney, Australia

^73^University of Adelaide, Adelaide, Australia
